# Early HIV treatment and survival over six years of observation in the ANRS 12249 Treatment as Prevention Trial

**DOI:** 10.1111/hiv.13263

**Published:** 2022-02-26

**Authors:** Kathy Baisley, Joanna Orne‐Gliemann, Joseph Larmarange, Melanie Plazy, Dami Collier, Jaco Dreyer, Thobeka Mngomezulu, Kobus Herbst, Willem Hanekom, Francois Dabis, Mark J. Siedner, Collins Iwuji

**Affiliations:** ^1^ Africa Health Research Institute Durban South Africa; ^2^ Faculty of Epidemiology and Population Health, London School of Hygiene and Tropical Medicine London UK; ^3^ University of Bordeaux Inserm Institut de Recherche pour le Développement (IRD) UMR 1219 Bordeaux France; ^4^ Centre Population et Développement (Ceped) Institut de Recherche pour le Développement (IRD) Université de Paris Inserm Paris France; ^5^ Division of Infection and Immunity University College London London UK; ^6^ Cambridge Institute of Therapeutic Immunology & Infectious Disease (CITIID) Cambridge UK; ^7^ DSI‐MRC South African Population Research Infrastructure Network Durban South Africa; ^8^ Harvard Medical School Boston Massachusetts USA; ^9^ Department of Global Health and Infection Brighton and Sussex Medical School University of Sussex Brighton UK

**Keywords:** HIV, immediate antiretroviral therapy, mortality, South Africa, test and treat

## Abstract

**Objectives:**

Population‐based universal test and treat (UTT) trials have shown an impact on population‐level virological suppression. We followed the ANRS 12249 TasP trial population for 6 years to determine whether the intervention had longer‐term survival benefits.

**Methods:**

The TasP trial was a cluster‐randomized trial in South Africa from 2012 to 2016. All households were offered 6‐monthly home‐based HIV testing. Immediate antiretroviral therapy (ART) was offered through trial clinics to all people living with HIV (PLHIV) in intervention clusters and according to national guidelines in control clusters. After the trial, individuals attending the trial clinics were transferred to the public ART programme. Deaths were ascertained through annual demographic surveillance. Random‐effects Poisson regression was used to estimate the effect of trial arm on mortality among (i) all PLHIV; (ii) PLHIV aware of their status and not on ART at trial entry; and (iii) PHLIV who started ART during the trial.

**Results:**

Mortality rates among PLHIV were 9.3/1000 and 10.4/1000 person‐years in the control and intervention arms, respectively. There was no evidence that the intervention decreased mortality among all PLHIV [adjusted rate ratio (aRR) = 1.10, 95% confidence interval (CI) = 0.85–1.43, *p* = 0.46] or among PLHIV who were aware of their status but not on ART. Among individuals who initiated ART, the intervention decreased mortality during the trial (aRR = 0.49, 95% CI = 0.28–0.85, *p* = 0.01), but not after the trial ended.

**Conclusions:**

The ‘treat all’ strategy reduced mortality among individuals who started ART but not among all PLHIV. To achieve maximum benefit of immediate ART, barriers to ART uptake and retention in care need to be addressed.

## INTRODUCTION

South Africa has the largest HIV epidemic in the world, with an estimated 7.5 million people living with HIV (PLHIV) and 5.2 million on antiretroviral treatment (ART) in 2019 [1]. Although HIV‐related mortality has reduced significantly with increased ART uptake, 22% of deaths in 2018 were attributable to HIV [2].

Clinical trials have shown the beneficial effect of early ART initiation on morbidity and mortality [3, 4]. Consequently, regardless of CD4 count, ART is now a global standard of care [5] and was adopted by South Africa in September 2016 [6]. However, the impact of this ‘treat all’ strategy on population‐level HIV mortality within programmatic settings is unknown. Several recent cluster‐randomized trials have demonstrated the potential of universal test and treat (UTT; i.e. the combination of universal testing campaigns and ‘treatment for all’) for population‐level impact [7]. The SEARCH trial in Uganda and Kenya found that UTT improved population‐level viral suppression and reduced mortality among PLHIV, but had no effect on HIV incidence [8]. By contrast, the MaxART trial in Eswatini found that ‘treat all’ improved population‐level viral suppression but had no impact on mortality [9].

The ANRS 12249 Treatment as Prevention (TasP) trial in rural South Africa compared immediate ART with standard‐of‐care (SOC) ART initiation. The trial found no effect on HIV incidence, although viral suppression was high among individuals who started ART [10]. The effect on mortality among PLHIV has not been examined. Although the TasP trial ended in June 2016, the cohort continued to be followed in the Africa Health Research Institute's (AHRI) demographic surveillance [11]. We undertook these analyses to address the question of whether a ‘treat all’ policy in rural South Africa provides long‐term survival benefits among PLHIV.

## METHODS

### Study design and setting

The TasP trial was a cluster‐randomized trial implemented by AHRI in KwaZulu‐Natal, South Africa [10, 12]. The trial was conducted in 22 commuities from March 2012 to June 2016. After the trial ended, the communities were included in the AHRI demographic surveillance area [11]. Households are surveyed up to three times annually to collect information on births, deaths and migration of all household members. When deaths are identified, a verbal autopsy interview is conducted with a close caregiver.

### Procedures

Trial procedures have been described previously [10, 12]. Briefly, all households were visited 6‐monthly by trained HIV counsellors. At each visit, residents aged ≥ 16 years were offered home‐based HIV counselling and testing (HBHCT) and were asked to provide a dried blood spot (DBS) sample for anonymous HIV testing for estimation of HIV incidence (trial's primary outcome). Those who accepted HBHCT were informed of their HIV diagnosis and referred to care at trial clinics in each community. Results from anonymous DBS testing were not returned to participants.

People living with HIV in the control arm were offered ART according to South African guidelines (CD4 < 350 cells/μL at trial start; CD4 < 500 cells/μL from January 2015). Those in the intervention arm were offered ART irrespective of CD4 count. The trial area was also served by three government clinics that provided ART according to national guidelines. PLHIV in both arms could opt to attend the government clinics or transfer to a trial clinic instead. At trial closure, participants were transferred to the government clinics where ART became available regardless of CD4 count from September 2016 [6].

Deaths during the trial were ascertained either by a tracing visit, if the participant was in care at a trial clinic and missed a visit, notification by a relative, or the 6‐monthly household visits. Deaths after the trial ended were ascertained through the AHRI demographic surveillance. The current study analysed all deaths through to 31 December 2018.

### Statistical methods

Data were collected using REDCap electronic data capture [13] and analysed using Stata 16.0 (College Station, TX, USA).

Our primary analysis was of all participants identified as HIV‐positive during the trial (through HBHCT, self‐report or anonymous DBS), to address the question of whether offering immediate ART reduces mortality among all PLHIV. We calculated person‐time from date of enrolment (if HIV‐positive at entry), date of first positive test (if status at entry was unknown), or date of seroconversion (if negative at entry and later tested positive). We estimated seroconversion dates as a random point between the last negative and first positive test, assuming a uniform distribution [14]. The exit date for the analysis was the earliest of date of death, out‐migration or last observed in the AHRI surveillance. Individuals who were not known to have out‐migrated or to have died but were not seen in the AHRI surveillance were censored administratively on 30 June 2016 (end of trial).

We estimated rate ratios (RRs) and 95% confidence intervals (CIs) for the effect of trial arm on mortality using random‐effects Poisson regression, to account for correlation within clusters. Crude RRs were adjusted for randomization stratum; adjusted RRs (aRRs) were adjusted for stratum, age, sex and period (during the trial, March 2012–June 2016; after trial end, July 2016–December 2018), to allow for temporal changes after trial closure. A period–treatment arm interaction term was included to allow the effect of trial arm to differ between periods.

The second analysis was restricted to PLHIV who were diagnosed (newly diagnosed during the trial or aware of their status at entry) and not on ART at trial entry, to examine whether offering immediate ART reduces mortality among PLHIV who are aware of their status but not yet on treatment. Person‐time was calculated as described earlier, beginning from the date of referral to clinic.

Lastly, we assessed the impact of offering early ART among individuals who started ART. We restricted it to those starting ART in the trial clinics in either arm, because deaths would be ascertained by the clinics. Person‐time was calculated from the date of starting ART.

For comparison, we examined mortality among HIV‐negative participants. Person‐time was calculated as described earlier, from date of the first HIV‐negative test. To allow estimation of the mortality rate, person‐time following the last negative test was considered negative for 2 years [15].

### Ethics

The TasP trial and cohort extension were approved by the Biomedical Research Ethics Committee (BFC 104/11 & BE335/19) of the University of KwaZulu‐Natal. The trial was also approved by the Medicines Control Council of South Africa.

## RESULTS

Overall, 8555 individuals aged ≥ 16 years were identified as HIV‐positive during the trial, of whom 87.4% (7474) were aware of their status. Among those aware, 5055 (67.6%) were not on ART. Baseline characteristics were reasonably balanced between arms for each of the three analytic subpopulations (Table 1). Among the 1865 individuals who started ART in the trial clinics, 1198 (64.2%) were ART‐naïve at entry. As expected, mean CD4 counts at initiation were higher in the intervention arm than in the control (451 vs. 379, *p* < 0.001).

**TABLE 1:** Baseline characteristics of each analysis group

	Total (*N* = 8555)	All people living with HIV		Aware of status and not on ART		Started ART during trial	
		Control (*N* = 4619)	Intervention (*N* = 3936)	Control (*N* = 2686)	Intervention (*N* = 2369)	Control (*N* = 912)	Intervention (*N* = 953)
	*N* (%)	*N* (%)	*N* (%)	*N* (%)	*N* (%)	*N* (%)	*N* (%)
Age at entry							
< 30 years	3432 (40.1%)	1835 (39.7%)	1597 (40.6%)	1187 (44.2%)	1072 (45.3%)	280 (30.7%)	370 (38.8%)
30–49 years	3899 (45.6%)	2111 (45.7%)	1788 (45.4%)	1170 (43.6%)	1004 (42.4%)	465 (51.0%)	421 (44.2%)
50+ years	1224 (14.3%)	673 (14.6%)	551 (14.0%)	329 (12.2%)	293 (12.4%)	167 (18.3%)	162 (17.0%)
Median (IQR)	32 (26–43)	33 (26–43)	32 (25–43)	31 (25–41)	31 (25–41)	36 (28–45)	33 (26–44)
Sex							
Male	2155 (25.2%)	1167 (25.3%)	988 (25.1%)	697 (25.9%)	626 (26.4%)	261 (28.6%)	273 (28.6%)
Female	6400 (74.8%)	3452 (74.7%)	2948 (74.9%)	1989 (74.1%)	1743 (73.6%)	651 (71.4%)	680 (71.4%)
Education							
Primary	3030 (35.4%)	1650 (35.7%)	1380 (35.1%)	893 (33.2%)	798 (33.7%)	418 (45.8%)	402 (42.2%)
Some secondary	3186 (37.2%)	1765 (38.2%)	1421 (36.1%)	1120 (41.7%)	921 (38.9%)	322 (35.3%)	350 (36.7%)
Secondary/above	2339 (27.3%)	1204 (26.1%)	1135 (28.8%)	673 (25.1%)	650 (27.4%)	172 (18.9%)	201 (21.1%)
Marital status							
Never married	6829 (79.8%)	3671 (79.5%)	3158 (80.2%)	2194 (81.7%)	1941 (81.9%)	742 (81.4%)	771 (80.9%)
Married/engaged	1427 (16.7%)	780 (16.9%)	647 (16.4%)	410 (15.3%)	356 (15.0%)	128 (14.0%)	153 (16.1%)
Divorced/separated/widowed	299 (3.5%)	168 (3.6%)	131 (3.3%)	82 (3.1%)	72 (3.0%)	42 (4.6%)	29 (3.0%)
Employment status							
Employed	1133 (13.2%)	605 (13.1%)	528 (13.4%)	395 (14.7%)	340 (14.4%)	120 (13.2%)	127 (13.3%)
Student	667 (7.8%)	366 (7.9%)	301 (7.6%)	233 (8.7%)	200 (8.4%)	27 (3.0%)	50 (5.2%)
Looking for work	2690 (31.4%)	1409 (30.5%)	1281 (32.5%)	820 (30.5%)	781 (33.0%)	273 (29.9%)	289 (30.3%)
Inactive/other	4065 (47.5%)	2239 (48.5%)	1826 (46.4%)	1238 (46.1%)	1048 (44.2%)	492 (53.9%)	487 (51.1%)
Stratum							
1	1100 (12.9%)	484 (10.5%)	616 (15.7%)	262 (9.8%)	311 (13.1%)	97 (10.6%)	127 (13.3%)
2	3444 (40.3%)	2039 (44.1%)	1405 (35.7%)	1182 (44.0%)	895 (37.8%)	462 (50.7%)	455 (47.7%)
3	4011 (46.9%)	2096 (45.4%)	1915 (48.7%)	1242 (46.2%)	1163 (49.1%)	353 (38.7%)	371 (38.9%)
On ART at trial start							
Yes	2419 (28.3%)	1372 (29.7%)	1047 (26.6%)	0	0	0	0
CD4 count at ART initiation							
Mean (SD)						379 (234.8)	451 (268.8)
Median (IQR)						343 (218–478)	411 (269–588)

Abbreviations: ART, antiretroviral therapy; IQR, interquartile range; SD, standard deviation.

We recorded 309 deaths among 8555 PLHIV. Median (IQR) follow‐up time was 3.79 (3.12–4.31) years, with 6614 (77.3%) individuals seen in the AHRI surveillance after the trial end. Crude mortality was 9.3/1000 and 10.4/1000 person‐years in the control and intervention arms, respectively, with no evidence of an effect of the TasP intervention (aRR = 1.10, 95% CI: 0.85–1.43, *p* = 0.46; Table 2; Figure S1). Mortality decreased among PLHIV in both arms in the period after the trial; however, there was no evidence that the effect of the intervention differed between periods (Table 2).

**TABLE 2:** Mortality among people living with HIV (PLHIV) in the TasP trial, overall and in period during the trial (March 2012–June 2016) and after the trial ended (July 2016–December 2018)

	Number individuals	Deaths/person‐years	Rate/1000 person‐years^a^	Crude RR (95% CI)^a^, ^b^	Adjusted RR (95% CI)^a^, ^c^
All PLHIV					
Overall				*p* = 0.44	*p* = 0.46
Control	4619	158/17 201	9.30	1	1
Intervention	3936	151/14 767	10.38	1.11 (0.86–1.43)	1.10 (0.85–1.43)
During trial^e^				*p* = 0.74	*p* = 0.83
Control	4619	107/9605	11.34	1	1
Intervention	3936	98/8304	11.96	1.05 (0.77–1.42)^d^	1.03 (0.76–1.41)^d^
After trial				*p* = 0.32	*p* = 0.28
Control	3632	51/7596	6.79	1	1
Intervention	2989	53/6463	8.36	1.22 (0.82–1.84)^d^	1.25 (0.83–1.88)^d^
PLHIV aware of status not on ART					
Overall				*p* = 0.40	*p* = 0.42
Control	2686	92/9526	9.64	1	1
Intervention	2369	92/8441	11.24	1.17 (0.81–1.70)	1.16 (0.81–1.67)
During trial^f^				*p* = 0.45	*p* = 0.52
Control	2686	53/5020	10.61	1	1
Intervention	2369	55/4510	12.57	1.19 (0.76–1.87)^d^	1.16 (0.74–1.79)^d^
After trial				*p* = 0.61	*p* = 0.54
Control	2160	39/4505	8.60	1	1
Intervention	1814	37/3931	9.76	1.14 (0.69–1.90)^d^	1.17 (0.71–1.93)^d^
PLHIV starting ART during the trial					
Overall				*p* = 0.07	*p* = 0.08
Control	912	52/3044	17.09	1	1
Intervention	953	39/3341	11.67	0.69 (0.45–1.04)	0.69 (0.45–1.04)
During trial^g^				*p* = 0.01	*p* = 0.01
Control	912	35/1561	22.43	1	1
Intervention	953	20/1820	10.99	0.49 (0.28–0.85)^d^	0.49 (0.28–0.85)^d^
After trial				*p* = 0.80	*p* = 0.69
Control	702	17/1483	11.46	1	1
Intervention	698	19/1522	12.49	1.10 (0.57–2.11)^d^	1.15 (0.59–2.21)^d^

Abbreviations: CI, confidence interval; RR, rate ratio.

^a^ Estimated from a Poisson regression model with random effects for community (cluster).

^b^ Adjusted for randomization stratum.

^c^ Adjusted for stratum, age and sex.

^d^ Estimated from model in footnotes 1–3, with and interaction between treatment arm and time period.

^e^ *p*‐value for interaction between treatment arm and period = 0.43.

^f^ *p*‐value for interaction between treatment arm and period = 0.97.

^g^ *p*‐value for interaction between treatment arm and period = 0.05.

Among 5055 PLHIV who were aware of their status and not on ART, we recorded 184 total deaths; crude mortality was 9.6/1000 (control) and 11.2/1000 (intervention) person‐years. Again, there was no evidence of an effect of the intervention on mortality (aRR = 1.16, 95% CI: 0.81–1.67, *p* = 0.42; Table 2), and no evidence that the effect differed between periods.

Among 1865 individuals starting ART at the trial clinics, mortality was lower in the intervention arm than in the control (11.7/1000 vs. 17.1/1000 person‐years, respectively), with a suggestion of benefit of the intervention overall (aRR = 0.69, 95% CI: 0.45–1.04, *p* = 0.08; Table 2). There was some evidence that the effect of the intervention differed between periods (*p*‐value for interaction = 0.05), with decreased mortality in the intervention arm during the trial (aRR = 0.49, 95% CI = 0.28–0.85, *p* = 0.01; Table 2), but not after the trial ended (aRR = 1.15, 95% CI = 0.59–2.21, *p* = 0.69).

Causes of death were recorded for 161 (52.1%) deaths (57 during trial and 104 after trial end). Nearly half (77, 47.8%) were attributed to infectious causes, 38 (23.6%) to non‐communicable diseases, 27 to unknown causes (16.8%) and 19 (11.8%) to other causes (mostly traumatic deaths including road traffic accidents and violence).

Crude mortality among HIV‐negative participants was 7.8/1000 person‐years (8.5/1000 during the trial and 6.5/1000 in the period after the trial).

## DISCUSSION

We found no evidence that offering immediate ART reduced mortality among all PLHIV in the TasP trial, or among those who were aware of their status but not on ART at trial entry, over 6 years of follow‐up. Among individuals who started ART, immediate ART decreased mortality considerably during the trial; however, that benefit was no longer evident after the trial ended.

Notably, in all analyses, mortality in both arms was relatively low, and decreased further after the trial ended, perhaps making it more difficult to detect an intervention impact. All‐cause mortality among PLHIV in the AHRI surveillance area in 2009–2011 was 36/1000 [16], decreasing to 23/1000 in 2011–2014 [15]. Our study shows a continued decline among PLHIV in more recent years, to around 8/1000 in 2016–2018. This probably reflects the general increase in ART availability, as South Africa implemented UTT and CD4 counts at ART initiation rose over time [17]. Mortality among PLHIV in our study was considerably lower than in the MaxART trial (21.6/1000 and 35.9/1000 in the control and intervention arms, respectively) [9], and similar to that in the SEARCH trial (control arm, 12.9/1000; intervention arm, 9.9/1000) [8].

Although clinical trials clearly demonstrate the benefit of early ART initiation for individuals [3, 4], the impact of the ‘treat all’ policy in routine practice in the public sector is less clear. The TasP trial differed from other UTT trials because it examined the impact of immediate ART only, with repeat universal testing offered to both arms. In the SEARCH trial, without repeat universal testing in the control arm, the UTT intervention decreased mortality among PLHIV by 23% after 3 years, despite having no effect on HIV incidence [8]. The authors attributed the mortality decline to a multi‐disease model of care, and accelerated ART initiation in individuals with low CD4 counts. By contrast, the MaxART trial examined immediate ART in the context of community HIV testing, and found no impact on mortality. Median follow‐up in MaxART was < 1 year; the authors suggested that longer follow‐up was needed to see an impact.

As expected, among individuals starting ART, we found a benefit of immediate ART on mortality during the trial, although this effect disappeared after the trial ended. The lack of an effect after the trial ended was driven primarily by a substantial decrease in mortality in the control arm (from 22.4/1000 during the trial to 11.5/1000 after the trial ended). CD4 counts at ART initiation were lower in the control arm; population studies in Africa have shown that mortality rates decline with increasing time on ART, and the decline is greatest in individuals starting ART at lower CD4 counts [18].

An underlying premise of UTT trials is that the intervention needs to address all steps in the HIV care cascade in order to reduce HIV transmission and, ultimately, mortality. Among TasP participants who were diagnosed but not on ART, only 47% linked to a trial clinic within 12 months [19]. A key lesson from the TasP trial was that the intervention failed to overcome barriers to linkage to care, resulting in low ART uptake. These results demonstrate that a UTT policy alone without attention to stigma and structural barriers is insufficient to improve population health outcomes.

Our study had limitations. Changes to national ART guidelines during the trial meant that the control arm became similar to the intervention arm, reducing the power to detect a difference between arms. We may have missed deaths during the trial, particularly among individuals who did not link to care; however, linkage was similar in both arms so any misclassification should be non‐differential. HIV status among all PLHIV may be misclassified because of a lack of confirmatory testing. Strengths include the large cohort, and ability to follow individuals through the AHRI demographic surveillance.

In summary, we found no evidence that immediate ART decreased mortality among all PLHIV over 6 years. Among individuals who started ART, immediate ART decreased mortality considerably during the trial, although the effect was no longer apparent after the trial ended. Our results and those of other studies clearly support the policy of early ART, but also highlight that failure to engage PLHIV in care can result in muted effects of the treat‐all policy. To achieve maximum benefit of immediate ART, the focus should be on addressing barriers to ART uptake and retention in care, tailored to understanding of the HIV epidemic in each setting.

## CONFLICT OF INTEREST

CI has received conference attendance support and research grant funding from Gilead Sciences unrelated to the submitted work. All other authors declare no conflicts of interest.

## AUTHOR CONTRIBUTIONS

KB: methodology, statistical analysis, writing original draft. JOG, FD: conceptualization, funding acquisition, writing original draft. JL, MS: methodology, review of original draft and editing. MP, DC, KH, WH: review of original draft and editing. JD: data management, review of original draft and editing. TM: data collection, review of original draft and editing. CI: conceptualization, funding acquisition, methodology, writing of original draft.

## Supporting information

Fig S1
