# Supplementary material for: Early HIV treatment and survival over six years of observation in the ANRS 12249 Treatment as Prevention Trial
Source: HIV Med. 2022 Feb 26;23(8):922–8. doi: 10.1111/hiv.13263 (PMC9545558; doi:10.1111/hiv.13263)

**Figure S1.** Kaplan-Meier plots of mortality in the 3 analysis subpopulations: all PHLIV (top); PLHIV aware of their status but not on ART (middle); PLHIV who started ART during trial.


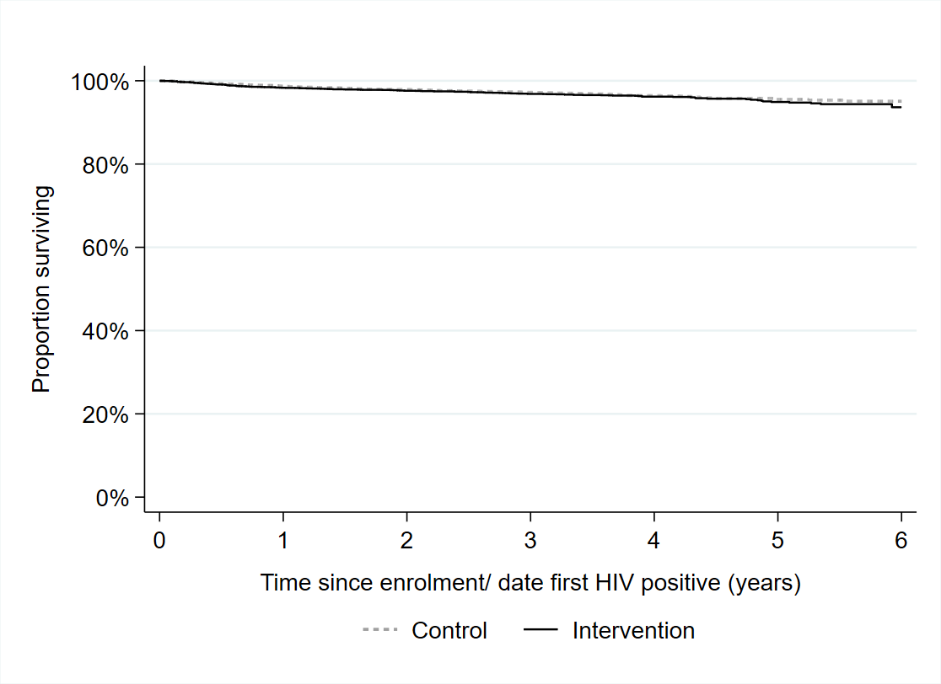


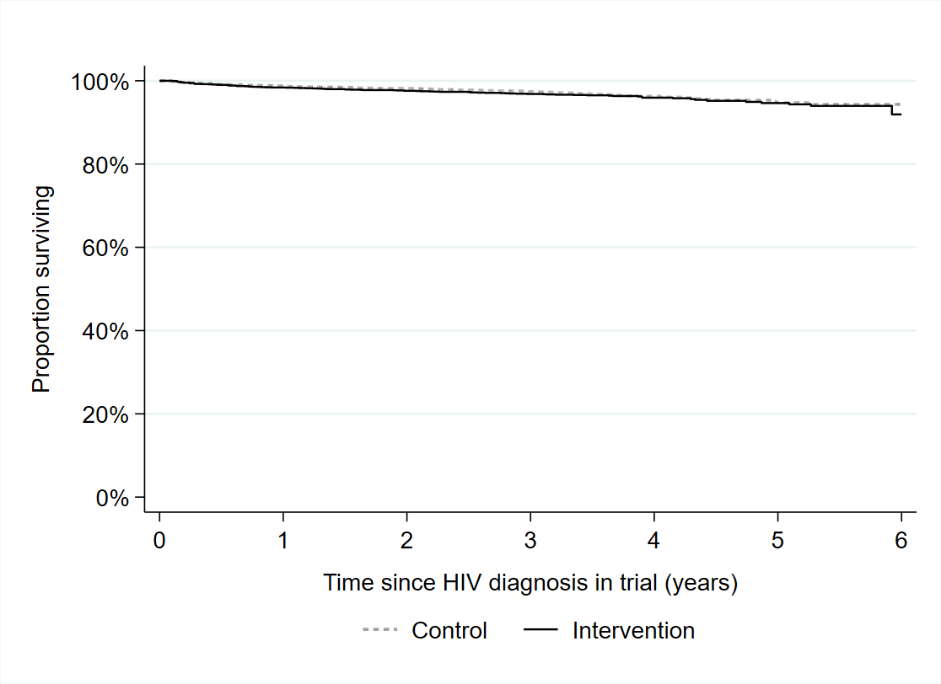


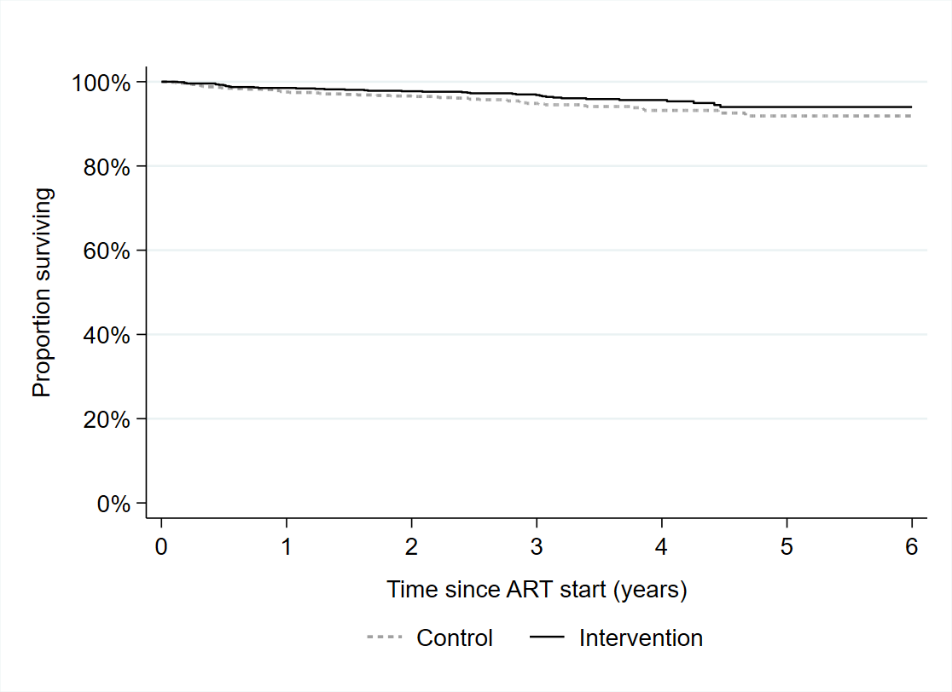

Supplement: Supplementary file 1 — Fig S1 [file HIV-23-922-s001.docx]
